# Therapeutic effects of metformin and clomiphene in combination with lifestyle intervention on infertility in women with obese polycystic ovary syndrome

**DOI:** 10.12669/pjms.331.11764

**Published:** 2017

**Authors:** Jing Zhang, Qinqin Si, Jinqiong Li

**Affiliations:** 1 Dr. Jing Zhang, Department of Obstetrics and Gynecology, Affiliated Heji Hospital of Changzhi College of Medicine, Changzhi 046000, P. R. China; 2 Dr. Qinqin Si, Department of Endocrinology, Affiliated Heji Hospital of Changzhi College of Medicine, Changzhi 046000, P. R. China; 3 Dr. Jinqiong Li, Department of Obstetrics and Gynecology, Affiliated Heji Hospital of Changzhi College of Medicine, Changzhi 046000, P. R. China

**Keywords:** Obesity, Clomiphene, Infertility, Lifestyle Intervention, Metformin, Polycystic ovary syndrome

## Abstract

**Objective:**

To evaluate the therapeutic effects of metformin and clomiphene in combination with lifestyle adjustment on infertility in women with obese polycystic ovarian syndrome (PCOS).

**Methods:**

A total of 101 infertile women with obese PCOS admitted to our hospital from July 2013 to July 2015 were randomly divided into an observation group (n=51) and a control group (n=50). The control group was treated with metformin plus clomiphene, based on which the observation group was also subjected to lifestyle adjustment. The body weight, body mass index (BMI) and waist-to-hip ratio (WHR) were measured before and after treatment. The changes of reproductive hormones, ovaries and endometrium were detected, and the rates of menstrual recovery, ovulation and pregnancy were observed.

**Results:**

The body weight and BMI of the observation group after treatment were significantly lower than those before treatment and of the control group (P<0.05). There was no significant difference in WHR between the two groups. In the observation group, there were significant differences in LH, T, LH/FSH, FINS and TG levels before and after treatment and from those of the control group after treatment (P<0.05). Both the left and right ovarian volumes of the observation group after treatment were significantly lower than those before treatment and of the control group after treatment (P<0.05). The menstrual recovery, ovulation and pregnancy rates of the observation group were significantly higher than those of the control group (P<0.05).

**Conclusion:**

Lifestyle adjustment combined with metformin and clomiphene can improve the reproductive endocrine and lipid metabolism of obese PCOS patients, decrease the volumes of left and right ovaries, and increase the menstrual recovery, ovulation and pregnancy rates.

## INTRODUCTION

Polycystic ovarian syndrome (PCOS) is an endocrine disorder with coexisting glucose metabolism abnormalities and reproductive dysfunction, with complex clinical symptoms as well as unclear etiology and pathogenesis. PCOS is clinically typified by androgen excess and chronic anovulation, manifested as menstrual disorders, hypomenorrhea, amenorrhea, obesity, infertility, hirsutism, acne, etc. At present, PCOS has mainly been associated with adrenal dysfunction, hypothalamic-pituitary dysfunction,^1^ insulin resistance, genetic factors, etc. In addition, the disease is often complicated by metabolic abnormalities of other tissues, increasing the risk of cardiovascular diseases and endometrial cancer. As a result, the life, mental and physical healths of patients are severely affected. Currently, PCOS is generally clinical treated by taking oral contraceptives and insulin, but the therapeutic effects remain unsatisfactory.^2^ This study assessed the effects of metformin and clomiphene in combination with lifestyle adjustment on infertility in obese PCOS patients by observing the changes of reproductive endocrine and lipid metabolism.

## METHODS

### Baseline clinical data

A total of 101 infertile women with obese PCOS admitted to our hospital from July 2013 to July 2015 were selected, who were aged 22-34 years old, with a mean age of (27.8 ± 4.6). The durations of infertility ranged from 2 to 5 years, (3.08 ± 0.81) on average. All patients were randomly divided into an observation group (n=51) and a control group (n=50). In the observation group, the patients were aged 23-35 years old, with a mean age of (28.3 ± 5.1). The durations of infertility ranged from 2 to 6 years, (3.54 ± 0.79) years on average. In the control group, the patients were aged 24-36 years old, with a mean age of (27.8 ± 5.4). The durations of infertility ranged from 2 to 5 years, (3.12 ± 0.78) years on average. The two groups had similar duration of infertility and age (P>0.05). This study has been approved by the ethics committee of our hospital, and written consent has been obtained from all patients.

### Diagnostic criteria

The criteria recommended by the European Society for Human Reproduction and Embryology and the American Society for Reproductive Medicine (ESHRE/ASRM) at the Rotterdam Conference in 2003 were used:^3^

1) Sporadic ovulation or anovulation
2) Clinical manifestations of hyperandrogenism and/or hyperandrogenemia
3) Ovarian polycystic changes: ≥12 follicles on one or both sides with ovarian diameters of 2-9 mm, and (or) ovarian volume ≥10 mL. Two of the three criteria were met, and other causes for hyperandrogenism, such as congenital adrenal hyperplasia, Cushing’s syndrome and tumors with androgen secretion, were excluded.

### Methods

The control group was started on orally taken metformin (Guizhou Tian’an Pharmaceutical Co., Ltd., National Medicine Permit No.H52020469) on the 1st-3rd days from menstruation or withdrawal bleeding, 500 mg/time, 3 times/day.^4^ It was taken after or at meal for three consecutive menstrual cycles. The patients began to additionally take clomiphene from the 5th day of the 4th menstrual cycle (Shanghai Hengshan Pharmaceutical Co., Ltd., National Medicine Permit No.H31021107), 50-100 mg/time, once/day. It was taken for 5 consecutive days and for 3 consecutive menstrual cycles.

The observation group received lifestyle adjustment on the basis of the control group:

1) ***Low-fat diet:*** Carbohydrates mainly included oats and ordinary Japanese rice, with oil intake limited and fat and fried foods avoided; proteins were mainly derived from plants, fish and shrimp.
2) ***Exercise strengthening:*** Exercise with unlimited forms was taken once (at least 30 min each time) in the morning and evening respectively and stopped when the patients began to sweat, which lasted for 6 consecutive months.
3) ***Smoking and drinking*** were forbidden, and the patients were followed up at regular intervals.

### Observation indices

1) The body weight, body mass index (BMI) and waist-to-hip ratio (WHR) were measured before and after treatment. BMI = Body mass (kg)/(height (m))^2^; WHR = waist circumference (cm)/hip circumference (cm).
2) Determination of reproductive hormones: Fasting venous blood was drawn on the 3rd-5th days of the menstrual cycle or during the early follicular phase by B-ultrasonography. The levels of luteinizing hormone (LH), testosterone (T), follicle-stimulating hormone (FSH), fasting insulin (FINS) and triglyceride (TG) were measured by immunofluorescence assay. The kits with eligible inner- and inter-batch errors were all purchased from Bayer Diagnostics Ltd. (Hong Kong, China).
3) The changes of ovaries and endometrium were detected by B-ultrasonography.
4) The menstrual recovery, ovulation and pregnancy rates of obese PCOS patients were observed six months after treatment.

### Statistical analysis

All data were analyzed by SPSS18.0, and subjected to one-way analysis of variance and variance homogeneity test. In the case of homogeneous variance, the LSD method was combined with the SNK method. The categorical data were expressed as (ax±s) and compared by the t test. The numerical data were compared by the χ^2^ test. P<0.05 was considered statistically significant.

## RESULTS

### Clinical therapeutic effects

There was no significant difference in WHR between the two groups. The body weight and BMI of the observation group after treatment were significantly lower than those before treatment and of the control group (P<0.05) (Table-I).

**Table-I:** Clinical therapeutic effects.

Group	n	Body weight (kg)		BMI (kg/m^2^)		WHR	

		Before treatment	After treatment	Before treatment	After treatment	Before treatment	After treatment
Observation	51	59.85±4.25	52.33±3.69^▲^	22.96±2.45	20.30±2.24^▲^	0.84±0.26	0.81±0.25
Control	50	60.09±4.13	57.35±3.86	23.36±2.36	22.12±2.32	0.86±0.24	0.83±0.24
t		0.689	7.557	0.691	4.403	0.359	0.625
T		>0.05	<0.05	>0.05	<0.05	>0.05	>0.05

Note:

▲ Compared with the data before treatment, P<0.05.

### Changes in reproductive endocrine and lipid metabolism indices

In the observation group, there were significant differences in LH, T, LH/FSH, FINS and TG levels before and after treatment (P<0.05). The control group had significantly different LH, T and LH/FSH levels before and after treatment (P<0.05). After treatment, the two groups also had significantly different LH, T, LH/FSH, FINS and TG levels (P<0.05) (Table-II).

**Table-II:** Changes in reproductive endocrine and lipid metabolism indices.

Group	LH (mIU/mL)	T (ng/mL)	LH/FSH	FINS (mIU/L)	TG (mmol/L)
*Observation (n=51)*					
Before treatment	16.52±5.58	3.02±0.82	1.92±0.28	37.85±7.65	1.89±0.23
After treatment	6.58±1.35^▲^*	0.95±0.13^▲^*	1.23±0.18^▲^*	20.21±4.30^▲^*	1.47±0.23^▲^*
*Control (n=50)*					
Before treatment	16.70±5.48	3.03±0.78	1.95±0.30	38.14±7.42	2.06±0.32
After treatment	7.03±1.41^▲^	1.25±0.20^▲^	1.42±0.20^▲^	30.89±4.21	1.82±0.22

Note:

▲ Compared with the data of the same group before treatment, P<0.05;

* compared with control group after treatment, P<0.05.

### Changes in ovaries and endometrium

Both the left and right ovarian volumes of the observation group after treatment were significantly lower than those before treatment and of the control group after treatment (P<0.05) (Table-III).

**Table-III:** Changes in ovaries and endometrium.

Group	n	Left ovarian volume (mL)		Right ovarian volume (mL)		Endometrial thickness (mm)	

		Before treatment	After treatment	Before treatment	After treatment	Before treatment	After treatment
Observation	51	8.56±2.56	5.63±1.25^▲^	8.32±2.62	5.45±1.09^▲^	6.79±2.60	6.35±2.39
Control	50	8.63±2.48	6.72±1.34	8.29±2.58	7.08±1.32	6.72±2.45	6.34±2.43
t		0.262	3.175	0.676	5.213	0.529	0.566
P		>0.05	<0.05	>0.05	<0.05	>0.05	>0.05

Note:

▲ Compared with the data of the same group before treatment, P<0.05.

### Menstrual recovery, ovulation and pregnancy rates

The menstrual recovery, ovulation and pregnancy rates of the observation group significantly exceeded those of the control group (P<0.05) (Table-IV).

**Table-IV:** Menstrual recovery, ovulation and pregnancy rates.

Group	n	Menstrual recovery rate	Ovulation rate	Pregnancy rate
Observation	51	48 (94.12)	40 (78.43)	18 (35.29)
Control	50	32 (64.00)	25 (50.00)	6 (12.00)
χ^2^		17.524	15.128	9.425
P	<0.05	<0.05	<0.05	<0.05

### Adverse reactions

At the beginning of medication, both groups suffered from gastrointestinal reactions such as nausea, vomiting and abdominal discomfort that disappeared after two weeks. During the first course of treatment, three cases of the observation group had small amounts of vaginal bleeding which stopped spontaneously.

## DISCUSSION

PCOS is the most common cause of menstrual disorders in women of reproductive age. The incidence of women in the reproductive period is about 5%-10%, but its etiology has not been fully clear yet.^5^ Endocrine disorders are the main clinical features of patients with PCOS, mainly manifested as increase of LH, LH/FSH and T at varying degrees, of which the reason may be the increased pituitary sensitivity to gonadotropin-releasing hormones, resulting in excessive LH secretion. Excessive LH can also stimulate theca cells and ovarian stroma to secret excessive androgen.^6^ Due to the above mechanisms, the ovarian androgen levels of PCOS patients were significantly higher than those of the normal women, and the follicle maturation process is inhibited, but the small follicles in the ovaries of patients can still secrete a certain amount of E2. At the same time, androstenedione can be transformed into estrone in peripheral tissues, and E2 and estrone can act on the hypothalamus and pituitary, whose positive feedback can lead to increased amplitude and frequency of LH secretion.^7^

The patients are sustained with high levels of LH, with no cyclical changes, so PCOS patients have no occurrence of anovulation. In addition, about 50% of PCOS patients also have varying degrees of insulin resistance, with a very complex mechanism. The molecular mechanism is mainly related to multiple aspects such as pre-receptor, receptor-level and post-receptor insulin resistance.^8^ The excess insulin in PCOS patients can act on the pituitary insulin receptor, enhance the release of LH and promote the adrenal gland and ovarian androgen secretion, so as to further aggravate the patients’ condition.^9^

About 40%-60% of PCOS patients have symptoms of obesity that is more prone to hyperinsulinemia and insulin resistance, among which the three are closely linked with each other.^8^ Therefore, the lifestyle should be adjusted at first, and attention paid to diet and exercise to help patients lose weight, reduce the indicators of blood insulin, triglyceride and testosterone, increase sex hormone-binding globulin, which are all conducive to the ovulation and conception of obese PCOS patients and reduce the rate of abortion.^10^-^12^ Decrease in BMI plays a favorable role in pregnancy outcomes and the improvement of endocrine and metabolic disorders.^13^ In this study, there was no significant difference in WHR between the two groups before and after treatment (P>0.05).

Since metformin was used to treat PCOS for the first time in 1994, many clinical studies have been performed. As an insulin-sensitive drug, metformin improves glucose metabolisms by promoting glucose absorption in the intestinal tract, reducing abnormal production of hepatic glycogen, facilitating anaerobic glycolysis and increasing glucose uptake and utilization by peripheral tissues such as muscles. As a result, the sensitivity to insulin is augmented, and the blood sugar level is decreased, accompanied by improved WHR.^14^ Meanwhile, insulin resistance-induced compensatory hyperinsulinemia was mitigated. Decrease in insulin level lowers those of LH and androgen as well as LH/FSH, helps correct hirsutism and acne, and recovers ovulatory menstruation.^15^ Palomba et al.^16^ found that the cumulative ovulation rate using metformin was similar to that using clomiphene, but the former led to higher rates of pregnancy and live birth.

In the observation group of metformin and clomiphene combined with lifestyle adjustment (including low-fat diet, exercise strengthening and forbidding of smoking and drinking), the weight and BMI after treatment were significantly lower than those before treatment and of the control group (metformin and clomiphene only) after treatment, among which the differences were statistically significant (P<0.05). Thus, the combination of metformin and clomiphene with lifestyle adjustment evidently reduced body weight, BMI and WHR. In the observation group, there were significant differences in LH, T, LH/FSH, FINS and TG levels before and after treatment (P<0.05).

In the control group, the differences in LH, T and LH/FSH were all significant between before and after treatment (P<0.05). After treatment, the two groups also had significantly different LH, T, LH/FSH, FINS and TG levels (P<0.05). Both the left and right ovarian volumes of the observation group after treatment were significantly lower than those before treatment and of the control group after treatment, with the differences being statistically significant (P<0.05). The menstrual recovery rate, ovulation rate and pregnancy rate of the observation group were significantly higher than those of the control group (P<0.05). Hence, for PCOS patients, the combination of medication with lifestyle adjustment had better effects than absolute medicine therapy, which could reduce body weight, regulate endocrine and metabolic disorders, restore menstruation to normal and improve ovulation rate.

In summary, lifestyle adjustment combined with metformin and clomiphene can significantly relieve insulin sensitivity of infertile patients with PCOS, lower BMI, regulate endocrine and metabolic disorders, recover menstruation to normal and elevate ovulation rate, as an ideal method for the treatment of PCOS-induced infertility.
